# The First Attempt to Reevaluate Radon and Thoron Exposure in Gansu Province Study Using Radon-Thoron Discriminating Measurement Technique

**DOI:** 10.3389/fpubh.2021.764201

**Published:** 2021-11-29

**Authors:** Hiromi Kudo, Shinji Yoshinaga, Xiaoliang Li, Shujie Lei, Shouzhi Zhang, Quanfu Sun, Chihaya Koriyama, Suminori Akiba, Shinji Tokonami

**Affiliations:** ^1^Graduate School of Health Sciences, Hirosaki University, Hirosaki, Japan; ^2^Research Institute for Radiation Biology and Medicine, Hiroshima University, Hiroshima, Japan; ^3^National Institutes for Quantum and Radiological Science and Technology, Chiba, Japan; ^4^National Institute for Radiological Protection, Chinese Center for Disease Control and Prevention, Beijing, China; ^5^Graduate School of Medical and Dental Sciences, Kagoshima University, Kagoshima, Japan; ^6^Kagoshima University, Kagoshima, Japan; ^7^Institute of Radiation Emergency Medicine, Hirosaki University, Hirosaki, Japan

**Keywords:** residential, radon, thoron, lung cancer, case-control study

## Abstract

Although the epidemiological studies provide evidence for an increased risk of lung cancer risk associated with residential radon, an issue of radon-thoron discrimination remains to be solved. In this study, an updated evaluation of lung cancer risk among the residents in Gansu, China was performed where one of the major epidemiological studies on indoor radon demonstrated an increased risk of lung cancer. We analyzed data from a hospital-based case-control study that included 30 lung cancer cases and 39 controls with special attention to internal exposure assessment based on the discriminative measurement technique of radon isotopes. Results from the analyses showed non-significant increased lung cancer risks; odds ratios (*OR*s) adjusted for age, smoking, and total income were 0.35 (95% *CI*: 0.07–1.74) and 0.27 (95% *CI*: 0.04–1.74) for groups living in residences with indoor radon concentrations of 50–100 Bq m^−3^ and over 100 Bq m^−3^, respectively, compared with those with < 50 Bq m^−3^ indoor radon concentrations. Although the small sample size hampers the usefulness of present analyses, our study suggests that reevaluation of lung cancer risk associated with residential radon in the epidemiological studies will be required on the basis of precise exposure assessment.

## Introduction

Uranium and radium are radioactive materials which are widely present in nature, particularly rocks and soils. Radon, thoron, and their progenies are generated from uranium and thorium, respectively, and they can be regarded as major contributors to exposures from the natural radiation sources. Radon is included in building materials and water. When breathing, radon and its progeny are unconsciously inhaled, they deposit in the lung and the tissues are exposed to alpha particles. This process can result in lung cancer (1). According to the UNSCEAR 2000 report (2), worldwide mean annual doses from radon, thoron, and their progenies are estimated to be 1.2 and 0.1 mSv a^−1^, respectively, which accounts for almost half of the total dose from natural radiation sources, i.e., 2.4 mSv a^−1^. The increased risk of lung cancer is demonstrated by the studies of underground miners who were exposed to the high levels of radon and its progeny (3, 4), these studies provide strong evidence for an association between radon and lung cancer. Since the 1980s, many case-control studies of lung cancer and residential radon exposure to persons have been conducted to investigate possible effects of relatively low levels of radon, but the study results are inconsistent mainly because of the small sample size of each study. For this reason, several meta-analyses and pooled analyses were subsequently conducted for Europe, North America, and China (1, 5–9). The European, North American, and Chinese pooling studies showed increased risks of lung cancer based on the measured radon concentration with an excess odds ratio (*OR*) per 100 Bq m^−3^ of 8% (95% *CI*: 3–16%), 11% (95% *CI*: 0–28%), and 13% (95% *CI*: 1%−36%), respectively. However, radon concentrations in some of these studies are likely to have been overestimated due to inability to discriminate between radon and thoron which could result in the biased estimates of lung cancer risk (10, 11). Thoron is present everywhere together with radon, and sometimes the radiation exposure due to thoron is at the same level or higher than that of radon (12). Therefore, it is important to consider the effects of not only radon and its progeny but also thoron and its progeny for precise evaluation of lung cancer risks. To address these issues, we conducted a hospital-based case-control study of the patients with lung cancer in Gansu Province, China, where an increased lung cancer risk was demonstrated in relation to radon by a large-scale study (13). In this paper, we report on the results of analyses of the initial set of data with special attention to exposure assessment by using the radon and thoron discriminative detectors; these have never been adopted in any previous epidemiological study of radon and lung cancer.

## Materials and Methods

### Study Area

This study was conducted by a hospital-based case-control design in four villages at Qingyang City, Gansu Province which is located on the Loess Plateau in northwestern China. More than 3 million people used to be living in cave dwellings in this area, and the residential mobility was quite low (14, 15). The typical cave dwelling consists of one room with a single entrance and two windows at the front side, which are built of local soil. The length, width, and height of the cave dwelling are typically 8, 3.3, and 3.3 m, respectively. These cave dwellings are equipped with a traditional bed, which is made of a loess brick (15).

### Study Subjects

The subjects included lung cancer cases who were diagnosed and confirmed with pathological evidence during the period from November 2005 to January 2007 in a major local hospital in the study area. Controls were selected with frequency-matching (2:1) from four villages in the study area, where age and sex distributions of the data were taken into account based on the result of the pilot survey. As a result, 33 cases and 72 controls were obtained. One or two trained nurses were allocated for each village of the study area so as to conduct an interview survey using a questionnaire. In brief, the questionnaire consisted of demographic characteristics, type of residence, residential history, medical history, educational background, marital status, and lifestyle factors, such as smoking and alcohol drinking. Out of the 105 subjects, 69 (65.7%) were male and 36 (34.3%) were female. Since the number of female lung cancer cases was too small (*n* = 3) while that of female controls was 33, the analyses in this study were restricted to data on 69 men (30 cases and 39 controls). Radon and thoron concentrations were measured for 82 dwellings, such as current and previous ones. When the radon, thoron, and, namely, equilibrium equivalent thoron concentration (EETC) concentration could not be measured in the previous dwellings, the average value of each current house type was used for the analysis. Two dwellings were not collected by the monitor. And the thoron progeny concentrations, EETC was also measured for these dwellings, but data on EETC were not available for 12 dwellings because of technical problems. As a result, the data analysis was conducted on 55 subjects (28 cases and 27 controls) for whom concentrations on radon, thoron, and EETC were all available.

### Measurements of Radon, Thoron, and Thoron Progenies

Residential radon and thoron were measured by using passive integrating radon-thoron discriminative monitor called RADOPOT (Radosys Co. Ltd., Hungary) (16, 17). The monitor used a CR-39 chip (BARYOTRAK, Nagase Landauer, Ltd., Japan) placed at the bottom. Furthermore, a long-term measurement of thoron progeny was conducted by using a monitor with CR-39 chip, which was developed by Zhuo and Iida (18) and was improved by Tokonami et al. (10). After the measurements were completed, the CR-39 chips were taken out of each monitor and were chemically etched for identifying alpha track reading system. The etching for CR-39 was carried out in a 6 M NaOH solution at 60°C for 24 h. We determined the track density through a track reading system such as an optical microscope. Using the track destiny and conversion factor, thoron progeny concentration could be obtained as the equilibrium equivalent thoron concentration. The two detectors were placed in the dwelling of each subject (case and control). One was set in the living area and the other in the sleeping area of the one-room dwellings. Each monitor hang from the ceiling in the center of the dwellings. The distance from the ceiling ranged from 5 to 30 cm (14). This protocol for placement of passive detectors was the same as the previous Gansu study (13). The measurements were carried out for 5–8 months in a batch starting from August to November 2006 and finishing in April 2007. The radon and thoron progeny concentrations were calculated as the average of the two detectors. We considered 5–30 years prior to the time of study entry as relevant exposure period related to lung cancer risk as in the other case-control studies. For the analysis, we used a time-weighted average of the radon concentrations in all the dwellings occupied over the exposure time window with weights proportional to the length of time the individual had lived in each dwelling. If the subject moved from the dwelling during the exposure period, the radon concentration was calculated by using a weighting coefficient for residence years. Weight for exposure time window (Wt) was defined as follows:

Wt1: the current dwelling
Wt2: the dwelling before the current dwelling

If the study participants have been living in the current dwelling for more than 30 years, Wt1 was 1 and Wt2 was 0. For more than 5 years and <30 years, Wt1 was the residence years minus 5 divided by 25, and Wt2 was 1 – Wt1. For <5 years, Wt1 was 0 and Wt2 was 1.

### Statistical Analysis

The descriptive statistics have been calculated for the demographic characteristics of the study subjects. Age and number of years smoking were compared by using the unpaired *t*-test for comparison between the cases and controls. Reference age, addiction history, drinking habits, resident type, current pre-detention, educational background, marital status the income, and occupation were compared by using the chi-square test. Unconditional logistic regression models were used to estimate the *OR*s) and 95% *CI*s according to radon concentration, with the lowest category as reference, adjusted for the potential confounders, such as age, smoking, and total income of household in 2004 as a socio economical factor. For the separated data on the case-control status and dichotomous or categorical covariates, Firth's method was applied in logistic regression. A test for trends in lung cancer risks was done by using the categorical scores (1, 2, and 3) for the groups based on radon concentrations. Statistical significance in the analysis was tested using two-sided *p*-values at a 5% level. The data analysis was implemented using IBM SPSS statistics 24.0 for Windows and SAS 9.4.

### Ethical Considerations

The survey was approved by the ethics review committee of Kagoshima University Graduate School of Medical and Dental Sciences on June 28, 2005 (No. 14).

## Results

### Background of the Subjects

The demographic and other characteristics of the study subjects (28 cases and 27 controls) were summarized in Table 1. The mean age of the subjects was 60.5 ± 9.3 (min 39–max 77) years old, and there was no significant difference (*p* = 0.64) between the cases (61.1 ± 8.6) and controls (59.9 ± 10.1). Almost all the subjects were current and former smokers, and the difference in smoking prevalence between the cases and controls was statistically significant (*p* = 0.008). The mean duration of smoking for cases and the controls were 35.2 ± 10.1 and 32.3 ± 9.9 years, respectively. As for drinking prevalence, there was no significant between the cases and controls (*p* = 0.10). The majority of cases were living in cave dwellings (*n* = 15, 53.6%) while only 10 controls (37.0%) were living in cave dwellings although this difference was not significant (*p* = 0.218). The mean years living in the current house were 20.7 ± 14.1 and 16.7 ± 17.3 years for cases and controls, respectively. Most subjects (*n* = 51, 92.7%) had lived in one or two dwellings for 6 months or more before moving to the current residence with no significant difference between the cases and controls (*p* = 0.83). With regard to educational background, most of the cases were educated at elementary or higher schools while controls were no school or illiterate, however, there was no significant difference (*p* = 0.77). We found five subjects (four cases and one control had a disease history of pulmonary tuberculosis, three of asthma (two cases and one control), nine (eight cases and one control) with chronic bronchitis, and two (both controls) with emphysema. Responders to the interview questionnaire were either the study subjects themselves or their next of kin.

**Table 1:** The demographic and other characteristics of the study subjects.

**Characteristic**	**Total**	**Cases**	**Controls**	***p*-value**
	**(*n* = 55)**	**(*n* = 28)**	**(*n* = 27)**	
Mean age, SD (years)	60.5 ± 9.3	61.1 ± 8.6	59.9 ± 10.1	0.645^a^
**Age groupings (years)**				
< 50	7 (12.7%)	3 (10.7%)	4 (12.0%)	0.285^b^
50–60	12 (21.8%)	4 (14.3%)	8 (28.0%)	
60–70	27 (49.1%)	17 (60.7%)	10 (40.0%)	
70>	8 (14.5%)	3 (10.7%)	5 (20.0%)	
No answer	1 (1.8%)	1 (3.6%)	0 (0.0%)	
**Smoking history**				
Current and former smokers	49 (89.1%)	28 (100%)	21 (77.8%)	0.008^b^
Never	6 (10.9%)	0 (0%)	6 (22.2%)	
Number of years smoking	34.0 ± 10.0	35.3 ± 10.1	32.3 ± 9.9	0.309^a^
**Drinking habits**				
Never	16 (29.1%)	8 (28.6%)	8 (29.6%)	0.101^b^
Only on festival days	26 (47.3%)	17 (60.7%)	9 (33.3%)	
Once a week	9 (16.4%)	2 (7.1%)	7 (25.9%)	
Every day	4 (7.3%)	1 (3.6%)	3 (11.1%)	
**Current residence type**				
Cave	25 (45.5%)	15 (53.6%)	10 (37.0%)	0.218^b^
Ordinary	30 (54.5%)	13 (46.4%)	17 (63.0%)	
**Duration of current residence (years)**				
Cave	31.7 ± 13.9	28.3 ± 13.2	37.4 ± 13.8	0.549^a^
Ordinary	8.3 ± 7.6	11.9 ± 9.3	5.6 ± 4.6	0.013^a^
**Duration of current residence (years)**				
< 5	13 (25.5%)	4 (15.4%)	9 (36.0%)	0.157^b^
5–29	29 (56.9%)	18 (69.2%)	11 (44.0%)	
30>	9 (17.6%)	4 (15.4%)	5 (20.0%)	
**Number of previous residences (for more than 6 months), before the current accommodation**				
0	1 (7.3%)	0 (7.1%)	1 (7.4%)	0.923^b^
1	32 (58.2%)	17 (60.7%)	15 (55.6%)	
More than 2	18 (34.5%)	9 (32.1%)	9 (37.0%)	
**Educational background**				
No schooling/illiterate	14 (27.5%)	4 (14.3%)	10 (40.0%)	0.077^b^
Elementary school	16 (29.0%)	12 (42.9%)	4 (16.0%)	
Junior high school	18 (31.9%)	10 (35.7%)	8 (32.0%)	
Others^c^	5 (8.7%)	2 (7.1%)	3 (12.0%)	
No answer	2 (2.9%)	0 (0.0%)	2 (5.1%)	
**Marital status**				
Never married	1 (1.8%)	0 (0.0%)	1 (3.7%)	0.080^b^
Married	48 (87.3%)	28 (100%)	20 (74.1%)	
Divorced	3 (5.5%)	0 (0.0%)	3 (11.1%)	
No answer	3 (5.5%)	0 (0.0%)	3 (11.1%)	
**Total income of household in 2004 (Renminbi)**				
Lower than 1,000 RMB	9 (15.7%)	8 (29.6%)	1 (4.0%)	0.0001^b^
1000– < 2000 RMB	14 (27.5%)	11 (42.3%)	3 (12.0%)	
2000– < 5000 RMB	11 (19.6%)	5 (19.5%)	6 (22.2%)	
5000– < 30000 RMB	29 (37.3%)	3 (11.5%)	16 (59.3%)	
**Occupation**				
Agricultural	15 (27.3%)	5 (17.9%)	10 (37.0%)	0.110^b^
Non-agricultural	40 (72.7%)	23 (82.1%)	17 (63.0%)	

a *Unpaired t-test*.

b *Chi-square test*.

c *Technical school/Senior high/polytechnic school/Junior college*.

### Radon, Thoron, and Thoron Progeny Exposure

The arithmetic means of radon concentration, thoron concentration, and EETC were 73.2 ± 48.6 Bq m^−3^ (ranged from 7.0 to 294.5), 275.4 ± 178.5 Bq m^−3^ (ranged from 9.7 to 1085), and 2.4 ± 1.1 Bq m^−3^ (ranged from 0.2 to 7.1), respectively. Their respective geometric means were 64.8, 225.5, and 2.4 Bq m^−3^. The radon concentration exceeded 100 Bq m^−3^ in 11 dwellings (20%) and 200 Bq m^−3^ in only one dwelling (1.8%). The arithmetic means of radon concentration, thoron concentration, and EETC for the cases were 61.8 ± 42.7 Bq m^−3^ (ranged from 7 to 199.5), 309.0 ± 220.6 Bq m^−3^ (ranged from 9.7 to 1085), and 2.3 ± 1.2 Bq m^−3^ (ranged from 0.2 to 5.4), respectively, while those for controls were 85.1 ± 52.2 Bq m^−3^ (ranged from 34.6 to 294.5), 240.7 ± 114.8 Bq m^−3^ (ranged from 69 to 581.5), and 2.6 ± 1.1 Bq m^−3^ (ranged from 1.0 to 7.1), respectively. As for radon concentration, thoron concentration, and EETC, there were no significant differences between the cases and controls (*p* = 0.08, *p* = 0.16, and *p* = 0.28). The arithmetic means of radon concentration, thoron concentration and EETC for the cave dwellings were 82.2 ± 46.6 Bq m^−3^ (ranged from 10 to 199.5), 210.5 ± 139.5 Bq m^−3^ (ranged from 9.7 to 630.5), and 2.5 ± 1.5 Bq m^−3^ (ranged from 0.2 to 7.1), respectively, while those for the ordinary type dwellings were 65.7 ± 49.7 Bq m^−3^ (ranged from 7 to 294.5), 329.5 ± 191.2 Bq m^−3^ (ranged from 69 to 1085), and 2.4 ± 0.7 Bq m^−3^ (ranged from 0.8 to 4.1), respectively. As for cave type dwellings, the thoron concentration was significantly lower than that of ordinary dwellings (*p* = 0.012), whereas the radon concentration and EETC were not significantly different between cave type dwelling and ordinary dwellings (*p* = 0.21 and *p* = 0.79). The radon concentration exceeded 100 Bq m^−3^ in nine cave house dwellings (36.0%), and none had a radon concentration of 200 Bq m^−3^. On the other hand, for ordinary type dwellings, the radon concentration exceeded 100 Bq m^−3^, in two dwellings (6.7%) and it was 200 Bq m^−3^ in one dwelling (3.3%). Table 2 shows the *OR*s in relation to measured radon concentrations adjusted for potential confounders. Indoor radon concentration was classified into three groupings of < 50, 50–100, and above 100 Bq m^−3^. This classification was done to keep a similar number of subjects in each grouping. The adjusted *OR*s for the groupings with indoor radon concentrations of 50–100 and above 100 Bq m^−3^ were 0.35 (95% *CI*: 0.07–1.74) and 0.27 (95% *Cl*: 0.04–1.74) for indoor radon concentrations of 50–100 and above 100 Bq m^−3^, respectively. There was no significant trend in lung cancer risk according to the radon concentration (*p* = 0.12). Similarly, indoor thoron progeny concentration was classified into three groupings of <1, 1–2, and above 2 Bq m^−3^. The adjusted *OR*s for the groupings with indoor thoron progeny concentrations of 1–2 and above 2 Bq m^−3^ were 0.43 (95% *CI*: 0.03–6.98) and 0.3 (95% *Cl*: 0.03–3.45) for indoor thoron progeny concentrations of 1–2 and above 2 Bq m^−3^, respectively. There was no significant trend in lung cancer risk according to the thoron progeny concentration (*p* = 0.29).

**Table 2:** Distributions of subjects and odds ratios (*OR*s) for lung cancer according to measured radon and thoron progeny concentration.

	**Cases**	**Controls**	**OR^a^**	**95% Cl**	***p* for trend**
**Radon concentration (Bq m** ^**−3**^ **)**					
< 50	13	6	1.00	Reference	0.14
50–100	11	14	0.35	0.07–1.74	
> 100	4	7	0.27	0.04–1.74	
**Thoron progeny concentration (Bq m** ^**−3**^ **)**					
< 1	4	1	1.00	Reference	0.29
1–2	7	4	0.43	0.03–6.98	
> 2	17	22	0.30	0.03–3.45	

a *Adjusted for reference age, smoking history, and total income of household in 2004*.

*OR, odds ratio; 95% Cl, 95% confidence interval*.

In the previous case-control study in Gansu, another passive radon monitor, called Radtrack, was used for exposure assessment. Radtrack is sensitive to thoron and a relative sensitivity of thoron is 0.68 when that of radon is normalized to be 1 (17). This means the reading will be 1.68 times higher than the actual radon concentration if radon concentrations are measured with this detector. Therefore, we calculated radon concentrations by assuming that the Radtrack detectors were used instead of RADPOT detectors. The overestimated radon concentration was calculated as follows:


(1) $$C_{Rn}\prime \left(Bq\;m^{-3}\right)=\;C_{Rn}\left(Bq\;m^{-3}\right)+0.68\;C_{Tn}\left(Bq\;m^{-3}\right)$$

Where C_*Rn*_ is radon concentration and C_*Tn*_ is thoron concentration. The arithmetic mean of the estimated radon concentration was 260.5 ± 125.7 Bq m^−3^ (ranged from 16.6 to 782.6). The estimated radon concentration dose was 3.6 times higher than our RADOPOT data. Table 3 shows the *OR*s adjusted for potential confounders. Exposure to indoor radon concentration was classified into three groupings of <200, 200–300, and above 300 Bq m^−3^. The adjusted *OR* of groupings with indoor radon concentration 200–300 and above 300 Bq m^−3^ were 0.31 (95% *CI*: 0.05–1.95), 0.54 (95% *CI*: 0.07–4.16), respectively, compared with the lower exposure grouping (199 Bq m^−3^). There was no significant increase in lung cancer risk based on overestimated radon concentration.

**Table 3:** Distribution of subjects and *OR*s for lung cancer according to overestimated radon concentration.

**Overestimated radon concentration (Bq m^**−3**^)**	**Cases**	**Controls**	**OR^a^**	**95% Cl**	***p* for trend**
< 200	9	5	1.00	Reference	0.66
200–300	11	16	0.31	0.05–1.95	
>300	8	6	0.54	0.07–4.16	

a *Adjusted for reference age, smoking history, and total income of household in 2004*.

*OR, odds ratio; 95% Cl, 95% confidence interval*.

## Discussion

To understand the lung cancer risk of exposure to low levels of residential radon, data from a case-control study were analyzed with special attention to internal exposure assessment. To the best of our knowledge, our present study is the first attempt in the world which used radon-thoron discriminative detectors to evaluate the exposure levels of radon and thoron separately and associated lung cancer risks. In the previous Gansu study (13), lung cancer risk significantly increased with increasing radon concentration, with an excess *OR* of 0.19 (95% *CI*: 0.05, 0.47) per 100 Bq m^−3^. In the present study, however, we observed no significant trend for lung cancer risk increase according to radon concentration. This apparent discrepancy could be explained by the low statistical power of the present study with the small number of cases and controls. Since the internal radiation dose could be a more important measure for evaluating the lung cancer risks than crude concentrations of radon and thoron, the annual effective dose derived from inhalation of radon (E_*RnP*_) and thoron (E_*TnP*_) progenies could be calculated as follows:


(2) $$\begin{array}{l} E_{RnP}\left(mSv\right)=C_{Rn}\left(Bq\;m^{-3}\right)\times 0.4\times 7000\left(h\right)\\ \;\times DCF_{RnP\;}\left(nSv{\left(Bq\;h\;m^{-3}\right)}^{-1}\right)\times 10^{-6} \end{array}$$

(3) $$\begin{array}{l} E_{Tnp}(mSv)=EETC\;(Bq\;m^{-3})\times 7000(h)\\ \;\times DCF_{Tnp}\left(nSv{\left(Bq\;h\;m^{-3}\right)}^{1}\right)\times 10^{-6} \end{array}$$

where C_Rn_ is radon concentration, the time spent in a dwelling is 7,000 h in a year, and DCF_*RnP*_ and DCF_*TnP*_ are dose conversion factors for radon progeny [9 nSv (Bq h m^−3^)^−1^] and thoron progeny [40 nSv (Bq h m^−3^)^−1^], respectively. These dose conversion factors for radon and thoron progeny were derived from the UNSCEAR 2006 report (4). The annual effective doses due to radon and thoron progenies were estimated to be 1.8 ± 1.2 (ranged from 0.2 to 7.4) mSv a^−1^, 0.7 ± 0.3 (ranged from 0.1 to 2.0) mSv a^−1^, respectively. The total dose was 2.5 ± 1.4 (ranged from 0.3 to 8.6) mSv a^−‘1^. On the other hand, ICRP 137 report (19) recommend that DCF_*RnP*_ and DCF_*TnP*_ are dose conversion factors for radon progeny [17 nSv (Bq h m^−3^)^−1^] and thoron progeny [107 nSv (Bq h m^−3^)^−1^], respectively. The annual effective doses due to radon and thoron progenies were estimated to be 3.5 ± 2.3 (ranged from 0.3 to 14) mSv a^−1^, 1.8 ± 0.9 (ranged from 0.1 to 5.3) mSv a^−1^, respectively. The total dose was 5.3 ± 2.7 (ranged from 0.6 to 17.1) mSv a^−1^.

The present study has shown that the thoron dose was as much as half of the radon dose. No attention has been paid to radiation dose from thoron in the terms of health effects, and there is no available epidemiological study of thoron (20). In Yang Jiang, one of the high natural background radiation areas of China, the annual effective doses are estimated to be 3.1 ± 2.0 mSv a^−1^ for radon and 2.2 ± 2.5 mSv a^−1^ for thoron (12). This means that the radon dose was comparable with that of thoron. Thus, thoron is no longer negligible from the viewpoint of health risk. Internal exposure situations in the high natural background radiation areas, such as Gansu should be clarified based on the precise exposure assessment. As shown in UNCSEAR report or ICRP publications, the effective dose coefficient for radon and thoron progenies are given. Therefore, radiation weighting factor of alpha particles 20 and tissue weighting factor of lung 0.12 were used for calculation of the absorbed dose for lung.

Therefore, we estimated the radon absorbed dose of the lung as follows:


(4) $$\begin{array}{r} D\left(mGy\right)=E÷WT÷WR \end{array}$$

Where E is the effective dose, WT is tissue weighting factor, the value is 0.12 and WR is radiation weighting factor the value is 20. If the dose conversion factor value with UNSCEAR 2006, the absorbed doses of lung due to radon and thoron progenies would be estimated to be 0.8 ± 0.5 (ranged from 0.01 to 3.1) mGy, 0.3 ± 0.1 (ranged from 0.02 to 0.8) mGy, respectively. The total dose was 1.0 ± 0.6 (ranged from 0.13 to 3.6) mGy. While we evaluated the absorbed doses of lung due to radon and thoron progenies with dose conversion factor from ICRP 137 report, the absorbed dose was 1.5 ± 1.0 (ranged from 0.14 to 5.8) mGy, 0.8 ± 0.4 (ranged from 0.06 to 2.2) mGy, respectively. The total dose was 2.2 ± 1.1 (ranged from 0.26 to 7.1) mGy.

Table 4-1 shows the *OR*s adjusted for potential confounders with conversion factor used UNSCEAR report. Exposure to absorbed doses was classified into three groupings of <1, 1–1.5, and above 1.5 mGy. The *OR* of groupings with total absorbed dose between 1 and 1.5, and above 1.5 mGy were 0.50 (95% *CI*: 0.11–2.34) and 0.48 (95% *Cl*: 0.08–2.98), respectively, compared with the lower exposure grouping (1 mGy). Same as above, Table 4-2 shows the *OR*s adjusted for potential confounders with conversion factor used ICRP 137 report. Exposure to the absorbed doses was classified into three groupings of <2, 2–3, and above 3 mGy. The *OR* of groupings with total absorbed dose between 2 and 3, and above 3 mGy were 0.26 (95% *CI*: 0.06–1.19) and 0.34 (95% *Cl*: 0.05–2.27), respectively, compared with the lower exposure grouping (2 mGy). In either case, there was no significant increase in lung cancer risk based on estimated radon, thoron absorbed dose.

**Table 4-1:** Distribution of subjects and *OR*s for lung cancer according to estimated radon, thoron absorbed dose (Using dose conversion factor derived from UNSCEAR).

**Total absorbed dose (mGy)**	**Cases**	**Controls**	**OR^a^**	**95% Cl**	***p* for trend**
< 1	20	13	1.00	Reference	0.33
1–1.5	5	9	0.50	0.11–2.34	
1.5>	3	5	0.48	0.08–2.98	

a *Adjusted for reference age, smoking history, and total income of household in 2004*.

*OR, odds ratio; 95% Cl, 95% confidence interval*.

**Table 4-2:** Distribution of subjects and *OR*s for lung cancer according to estimated radon, thoron absorbed dose (Using dose conversion factor derived from ICRP137).

**Total absorbed dose (mGy)**	**Cases**	**Controls**	**OR^a^**	**95% Cl**	***p* for trend**
< 2	18	9	1.00	Reference	0.14
2–3	7	13	0.26	0.06–1.19	
3>	3	5	0.34	0.05–2.27	

a *Adjusted for reference age, smoking history, and total income of household in 2004*.

*OR, odds ratio; 95% Cl, 95% confidence interval*.

We measured residential radon concentration by using the passive integrating radon-thoron discriminative monitor. The arithmetic means of radon concentration, for the cases was 62.1 ± 43.4 Bq m^−3^ (ranged from 7 to 199.5), while for controls was 82.4 ± 54.6 Bq m Table^−3^ (ranged from 1 to 294.5), respectively. According to the previous study by Wang et al. (13) in the same study area, mean radon concentrations were 230.4 Bq m^−3^ for the cases, and 222.2 Bq m^−3^ for controls. In the present study, the arithmetic means of radon concentration were fourth in the cases and third in controls compared with the results of Wang et al. study which were likely to be related to overestimation by the radon-thoron discrimination issue. Our study area is located on the Loess Plateau in China, and many people ever lived in cave type dwelling which made of a loess, for many years. The cave type house, due to its low ventilation rate and high exhalation rate of radon (loess walls are not painted or not well-painted) (15), and the radon level was high. Shang et al. (21) surveyed radon, and thoron and its decay products in the Chinese traditional residential dwellings constructed of loam bricks or soil walls, and they found the radon concentration was 72.4 ± 59.2 Bq m^−3^, thoron concentration was 318 ± 368 Bq m^−3^, and thoron progeny concentration was 3.8 ± 3.3 Bq m^−3^ with a maximum value of 15.8 Bq m^−3^. According to the radon and thoron measurements for cave type houses carried out in Luliang and Yan'an in China (15), the radon concentration was 55Bq m^−3^ (ranged from 17 to 179). Thoron concentration was 148 Bq m^−3^ (ranged from 10 to 760) and thoron progeny concentration was 1.5 Bq m^−3^ (ranged from 17 to 179). These results and those of the present study suggested that the mean radon concentration was unlikely to exceed 100 Bq m^−3^ in this same and nearby areas. In recent years, some papers reported discriminative monitors have been used for long-term measurements of indoor radon and thoron (22–25). In addition, thoron progeny concentration was measured (26–30). The reported radon concentration ranged from a few 10 to over 100 Bq m^−3^. On the other hand, from the dosimetric point of view, thoron progeny concentration was somewhat higher than that of radon progeny. According to Cameroon study (29), thoron progeny contribution to inhalation dose was found to be 30%. Some papers showed that thoron was probably more important contribution to inhalation dose (27, 30). A further study is required to acquire correct radon concentrations and to better evaluate the risk of lung cancer. Future prospects, in terms of epidemiological studies on residential radon and lung cancer, measured radon-222 (radon) concentrations might be affected by radon-220 (thoron) signals. It is well-known that radon-220 concentrations vary with space, especially distance from the sources, such as building materials (31). According to Kovacs (22), summarized radon and thoron survey in Hungary, despite the radon-thoron discriminative monitor set the distance of 15–20 cm from the wall, thoron concentrations were exceeding the detection level. Therefore, the presence of thoron needs to be considered unless a strict protocol on the proper placement of radon monitors is followed. As long as the radon concentration data were accurate and the measuring devices were carefully used in the Chinese, American, and European studies, the interference of thoron in radon measurements cannot be ignored. Precise assessment of radon concentration in such epidemiological studies is required in the future for more reliable estimates of radon risk as well as thoron risk. According to Tokonami (32), in the calculation of indoor annual effective dose for radon, an equilibrium factor was used. However, equilibrium factor for thoron is not applicable or meaningful because thoron varies drastically in space. Therefore, the lung cancer risk must not be assessed with thoron concentrations. On the other hand, the risk should be assessed with thoron progeny concentrations. This is because thoron progeny concentrations are constant regardless of the distance from the wall (31). As we repeated, thoron concentrations should not be used for lung cancer risk analysis, but they need to be measured to determine radon concentration accurately by eliminating thoron interference. As long as, thoron is present together with radon, thoron itself cannot be ignored unless it is directly measured. From the dosimetric point of view, the dose derived from thoron progeny inhalation needs to be evaluated under such exposure situations where both radon and thoron are present. It will be much difficult to conduct such an epidemiological survey in Gansu Province, China because many residents moved from caves in the countryside to detached houses in the city. However, if a new epidemiological study is further initiated somewhere, the proposed methodology will help more reliable data collection.

## Data Availability Statement

The original contributions presented in the study are included in the article/supplementary material, further inquiries can be directed to the corresponding authors.

## Ethics Statement

The studies involving human participants were reviewed and approved by Kagoshima University Graduate School of Medical and Dental Sciences. The patients/participants provided their written informed consent to participate in this study.

## Author Contributions

All authors listed have made a substantial, direct, and intellectual contribution to the work and approved it for publication.

## Funding

This research was funded by the KAKENHI, Japan Society for the Promotion of Science (JSPS), grant numbers 17406002, 16K15368, 18KK0261, and 20H00556.

## Conflict of Interest

The authors declare that the research was conducted in the absence of any commercial or financial relationships that could be construed as a potential conflict of interest.

## Publisher's Note

All claims expressed in this article are solely those of the authors and do not necessarily represent those of their affiliated organizations, or those of the publisher, the editors and the reviewers. Any product that may be evaluated in this article, or claim that may be made by its manufacturer, is not guaranteed or endorsed by the publisher.

## References

[1] WHO Guidelines Approved by the Guidelines Review Committee. WHO Handbook on Indoor Radon: A Public Health Perspective, World Health Organization. Geneva: World Health Organization (2009). 23762967

[2] United Nations Scientific Commitee on the Effects of Atomic Radation. Sources and Effects of Ionizing Radiation, United Nations Scientific Commitee on the Effects of Atomic Radation, UNSCEAR 2000 Report to the General Assembly With Scientific Annexes. New York, NY: United Nations (2000).

[3] Council N. R. Health Effects of Exposure to Radon: BEIR VI, Vol. 6. Washington, DC: National Academies Press (1999). 25121310

[4] United Nations Scientific Commitee on the Effects of Atomic Radation. Sources and Effects of Ionizing Radiation, United Nations Scientific Commitee on the Effects of Atomic Radation, UNSCEAR 2006 Report to the General Assembly With Scientific Annexes, Volume II. New York, NY: United Nations (2006).

[5] Darby S Hill D Auvinen A Barros-Dios J Baysson MH Bochicchio F. Radon in homes and risk of lung cancer: collaborative analysis of individual data from 13 European case-control studies. BMJ. (2005) 330:223. 10.1136/bmj.38308.477650.63 15613366 PMC546066

[6] Darby S Hill D Deo H Auvinen A Barros-Dios JM Baysson H. Residential radon and lung cancer—detailed results of a collaborative analysis of individual data on 7148 persons with lung cancer and 14208 persons without lung cancer from 13 epidemiologic studies in Europe. Scand J Work Environ Health. (2006) 32:1–84. 16538937

[7] Krewski D Lubin JH Zielinski JM Alavanja M Catalan VS Field RW. Residential radon and risk of lung cancer: a combined analysis of 7 North American case-control studies. Epidemiology (Cambridge, Mass). (2005) 16:137–45. 10.1097/01.ede.0000152522.80261.e3 15703527

[8] Krewski D Lubin JH Zielinski JM Alavanja M Catalan VS William Field R. A combined analysis of North American case-control studies of residential radon and lung cancer. J Toxicol Environ Health Part A. (2006) 69:533–97. 10.1080/15287390500260945 16608828

[9] Lubin JH Wang ZY Boice JD Jr. Xu ZY Blot WJ De Wang L. Risk of lung cancer and residential radon in China: pooled results of two studies. Int J Cancer. (2004) 109:132–7. 10.1002/ijc.11683 14735479

[10] Tokonami S. Why is 220Rn (thoron) measurement important? Radiat Prot Dosimetry. (2010) 141:335–9. 10.1093/rpd/ncq246 20846967

[11] Doi K Tokonami S Yonehara H Yoshinaga S. A simulation study of radon and thoron discrimination problem in case-control studies. J Radiat Res. (2009) 50:495–506. 10.1269/jrr.09054 19680008

[12] Kudo H Tokonami S Omori Y Ishikawa T Iwaoka K Sahoo SK. Comparative dosimetry for radon and thoron in high background radiation areas in China. Radiat Prot Dosimetry. (2015) 167:155–9. 10.1093/rpd/ncv235 25935013

[13] Wang Z Lubin JH Wang L Zhang S Boice JD Jr Cui H. Residential radon and lung cancer risk in a high-exposure area of Gansu Province, China. Am J Epidemiol. (2002) 155:554–64. 10.1093/aje/155.6.554 11882529

[14] Yamada Y Sun Q Tokonami S Akiba S Zhuo W Hou C Zhang S. Radon–thoron discriminative measurements in Gansu Province, China, and Their implication for dose estimates. J Toxicol Environ Health Part A. (2006) 69:723–34. 10.1080/15287390500261265 16608835

[15] Sun Q Tokonami S Hou C Zhang H S Akiba S Zhuo W. Epidemiological potentials of radon-and thoron-prone area in China. Jpn J Health Phys. (2004) 39:257–62. 10.5453/jhps.39.257

[16] Zhuo W Tokonami S Yonehara H Yamada Y. A simple passive monitor for integrating measurements of indoor thoron concentrations. Rev Sci Instrum. (2002) 73:2877–81. 10.1063/1.149323324313031

[17] Tokonami S Zhuo W Ryuo H Yonehara H Yamada Y Shimo M. Instrument performance of a radon measuring system with the alpha-track detection technique. Radiat Prot Dosimetry. (2003) 103:69–72. 10.1093/oxfordjournals.rpd.a006118 12596992

[18] Zhuo W Iida T. Estimation of thoron progeny concentrations in dwellings with their deposition rate measurements. Jpn J Health Phys. (2000) 35:365–70. 10.5453/jhps.35.365

[19] ICRP Publication 137. Occupational intakes of radionuclides: part 3.1. Ann ICRP. (2018) 46:1–486. 10.1177/0146645317734963 29380630

[20] United Nations Scientific Committee on the Effects of Atomic Radiation. Sources and Effects of Ionizing Radiation, United Nations Scientific Committee on the Effects of Atomic Radiation, UNSCEAR 2019 Report to the General Assembly with Scientific Annexes, Volume I. New York, NY: United Nations (2019).

[21] Shang B Chen B Gao Y Wang Y Cui H Li A. Thoron level in traditional Chinese residental dwellings. Radiat Environ Biophys. (2005) 44:193–9. 10.1007/s00411-005-0020-5 16283349

[22] Kovacs T. Thoron measurements in Hungary. Radiat Prot Dosimetry. (2010) 141: 328–34. 10.1093/rpd/ncq232 20966202

[23] Phon LK Dung BD Chau ND Kovacs T Nam NV Hao DV. Estimation of effective dose rates caused by radon and thoron for inhabitants living in rare earth field in northwestern Vietnam (Lai Chau province). J Radioanal Nucl Chem. (2015) 306:309–16. 10.1007/s10967-014-3881-8

[24] Adelikhah M Shahrokhi A Imani M Chalupnik S Kovacs T. Radiological assessment of indoor radon and thoron concentrations and indoor radon map of dwelling in Mashhad Iran. Int J Environ Res Public Health. (2021) 18:141. 10.3390/ijerph18010141 33379145 PMC7794745

[25] Chen J Bergman L Falcomer R Whyte J. Result of simulations radon and thoron measurement in 33 metropolitan areas of Canada. Radiat Prot Dosimetry. (2015) 163:210–6. 10.1093/rpd/ncu141 24748485 PMC4312419

[26] Skeppström K Wåhlin E. Is thoron a problem in Swedish dwellings? results of measurements of concentrations of thoron and its progeny. Radiat Prot Dosimetry. (2015) 167:107–10. 10.1093/rpd/ncv212 25904697

[27] Smetsers RCGM Blaauboer RO Dekkers F Slaper H. Radon and thoron progeny in dutch dwellings. Radiat Prot Dosimetry. (2018) 181:1–14. 10.1093/rpd/ncy093 29931357

[28] Žunić ZS Stojanovska Z Veselinović N Mishra R Yarmoshenko IV Sapra BK. Indoor radon, thoron and their progeny concentrations in high thoron rural Serbia environments. Radiat Prot Dosimetry. (2017) 177:36–9. 10.1093/rpd/ncx167 29036675

[29] Nkoulou JEN Engola LN Saïdou Hosoda M Bongue D Susuki T. Simultaneous indoor radon, thoron and thoron progeny mesumerent in Betare-Oya gold mining areas, Eastern Cameroon. Radiat Prot Dosimetry. (2019) 185:391–401. 10.1093/rpd/ncz026 30916308

[30] With GD Smetsers RCGM Slaper H Jong PD. Thoron exposure in Dutch dwellings—an overview. J Environ Radioact. (2018) 183:73–81. 10.1016/j.jenvrad.2017.12.014 29306710

[31] Hosoda M Kudo H Iwaoka K Yamada R Suzuki T Takakuma Y. Characteristics of thoron (^220^Rn) in environment. Appl Rad Isotopes. (2017) 120:7–10. 10.1016/j.apradiso.2016.11.014 27894046

[32] Tokonami S. Characteristics of thoron (^220^Rn) and its progeny in the indoor environment. Int J Environ Res Public Health. (2020) 17:8769. 10.3390/ijerph17238769 33255858 PMC7728306

